# Reward Associated with Singing Behavior Correlates with Opioid-Related Gene Expression in the Medial Preoptic Nucleus in Male European Starlings

**DOI:** 10.1371/journal.pone.0115285

**Published:** 2014-12-18

**Authors:** Lauren V. Riters, Sharon A. Stevenson, M. Susan DeVries, Melissa A. Cordes

**Affiliations:** Department of Zoology, University of Wisconsin Madison, 428 Birge Hall, Madison, Wisconsin, United States of America; Oregon Health and Science University, United States of America

## Abstract

Birdsong consists of species-specific learned vocal sequences that are used primarily to attract mates and to repel competitors during the breeding season. However, many birds continue to sing at times when vocal production has no immediate or obvious impact on conspecific behavior. The mechanisms that ensure that animals produce important behaviors in contexts in which the function of these behaviors is not immediate or obvious are not known. One possibility is that animals engage in such behaviors because they are associated with pleasure. Here we examined the hypothesis that male European starlings sing outside of the breeding season in part because the act of singing in this context is facilitated and/or maintained by opioid-mediated reward. We measured song-associated reward using a conditioned place preference (CPP) test in male starlings producing fall, non-breeding season-typical song. We used quantitative real time PCR to measure expression of the enkephalin opioid precursor preproenkephalin (PENK) and mu opioid receptors (MOR) in the medial preoptic nucleus (POM; a region in which opioids are implicated in both reward and starling fall song) and additionally the song control region HVC as a control. Starlings developed a strong preference for a place that had been paired previously with the act of producing fall-typical song, indicating that fall song production was associated with a positive affective state. Both PENK and MOR mRNA expression in the POM, but not HVC, correlated positively with both individual reward state (as reflected in CPP) and undirected singing behavior. These results suggest that singing induces opioid receptor and enkephalin expression in the POM and consequent reward, and/or that opioid release in the POM induced by individual or environmental factors (e.g., the presence of food, safety of a flock or the absence of predators) induces a positive affective state which then facilitates singing behavior.

## Introduction

Animals commonly produce behaviors that result in immediate, observable endpoints. For example, nesting animals collect grass to build nests, predators hunt to acquire food, and animals swim, fly or run to reach goal locations. However, there are situations in which the immediate function of behavior is not clear. Examples include various forms of play behavior in young animals as well as vocal behavior produced in the absence of any apparent recipient(s). Although engaging in behavior with no obvious or immediate function may play important roles in learning and maintaining the behavior (review related to play behavior [1]), the factors that reinforce such behaviors are not clear. One possibility is that animals engage in such activities because they are intrinsically rewarding and/or facilitated by an intrinsic reward state [2]–[5]. The present study was designed to explore this hypothesis in a study of singing behavior in male European starlings, *Sturnus vulgaris*.

Birdsong consists of repertoires of species-specific learned vocal sequences that are used primarily to attract mates and to repel competitors [6]. These types of conspecific-directed songs can be reinforced immediately through copulation or the departure of a rival (reviewed in [3]–[5]). In addition to producing conspecific-directed songs, songbirds also sing in contexts in which song does not appear to evoke any obvious (at least to a human observer), immediate behavioral response in other individuals [7]–[12]. This distinction between song that is directed towards a conspecific for an immediate purpose and song that is not was noted by Charles Darwin who suggested that, “[although] the songs of birds serve mainly as an attraction during the season of love (p. 662), male birds…continue singing for their own amusement after the season for courtship is over (p. 370)” [2]. Although song outside the context of courtship may not have any obvious, direct effects, it does have clear benefits. By repeating and varying singing behavior outside of its primary context, birds improve the effectiveness of song during future interactions with mates or rivals. Furthermore, this type of singing behavior may maintain group cohesion or social order [9], [13]; however, there does not appear to be any obvious, immediate reinforcer, suggesting that much like well-studied play behaviors in rats [1], birds may sing in these contexts because it is highly rewarding and/or facilitated by an already experienced positive affective state.

Recent converging data support the hypothesis that conspecific-directed song is reinforced by the consequences it has on receiver behavior; whereas, nonconspecific-directed (or undirected) song is reinforced and/or stimulated at least in part by “pleasure.” [14]. Specifically, the rewarding properties associated with singing undirected and conspecific-directed song were tested in both male European starlings and zebra finches, *Taeniopygia guttata* using a conditioned place preference paradigm (a classic method used to evaluate the rewarding properties of a behavior or stimulus, such as food, sexual behavior or the use of drugs of abuse [15]–[17]). Both male starlings and zebra finches developed a preference for a place that had been paired previously with the act of producing undirected song but not conspecific-directed song [14]. This place preference (i.e., measure of reward state) was positively linearly correlated with undirected (but not conspecific-directed) singing behavior, offering the first evidence for a tight coupling between the act of producing undirected song and reward.

Mechanisms underlying reward have been well studied with respect to feeding, sexual behavior, and drug use. These studies demonstrate that both dopamine and opioid neuropeptides are involved in the production of behaviors that result in reward, with dopamine underlying motivation and the pursuit of a reward and opioids underlying pleasure induced by the acquisition of a reward [18]–[23]. Consistent with a role for dopamine in motivational aspects of singing behavior, both dopamine and dopaminergic markers in the ventral tegmental area (VTA) and VTA projection regions are tightly coupled to song that is used by males to attract females [24]–[27]. Furthermore dopamine in the songbird striatum (area X) is released at higher levels during production of conspecific-directed compared to undirected song [28]. Dopamine may also play a role in undirected song (e.g., [29]); however, a growing number of studies strongly implicates opioid neuropeptides in facilitating or maintaining undirected singing behavior. For example, systemic injections of the nonselective opioid receptor antagonist naloxone suppressed undirected singing behavior in male zebra finches [30]. Furthermore, in male starlings undirected (but not female-directed) singing behavior was positively correlated with analgesia, an indirect measure of opioid release [31].

Data suggest the medial preoptic nucleus (often referred to as POM in birds) as at least one site in which opioids may underlie reward associated with the production of undirected song. Since the late 1970s, opioid activity has been proposed to mediate positive social affect in vertebrates (i.e., reward associated with social contact or affiliative social interactions) ([32]–[34] and reviewed in [35]), and the preoptic nucleus was one of the first brain regions in which opioids were proposed to mediate prosocial interactions in rodents [36]. Opioid activity, including mu opioid receptor stimulation and enkephalins, in the preoptic area, induces reward in rats [37], [38]. In male starlings, met-enkephalin immunolabeling measures in the POM correlated positively with undirected but not female-directed song [39]. Additionally, mu opioid receptor measures in the POM related to undirected song in a curvilinear (inverted-U shaped) manner, such that both low and high singers had relatively low densities of mu opioid receptor label [40]. Because, opioid receptors internalize and down-regulate in response to high concentrations of ligand [41], [42], this finding is consistent with the possibility that high met-enkephalin release caused receptor down-regulation in the highest singing animals.

When considered together, these separate studies suggest undirected singing behavior, reward state, and opioid markers are tightly coupled; however, relationships among these variables have not been examined in a single set of birds, and to date studies have focused only on opioid-related proteins measured using immunohistochemistry. The goal of the present study was to more directly characterize these relationships by determining the extent to which mRNA expression for opioid-related markers in the POM statistically predicted undirected song-associated reward in a single set of male European starlings producing undirected song in fall-typical, non-breeding condition flocks. To do this, we measured song-associated reward using a conditioned place preference (CPP) test (as in [14]). We then collected neural tissue and used quantitative real time PCR (qPCR) to measure expression of the enkephalin opioid precursor preproenkephalin (PENK) and mu opioid receptors (MOR) in the POM and additionally the song control region HVC as a control.

## Materials and Methods

### Animals

This study was carried out in strict accordance with the recommendations in the Guide for the Care and Use of Laboratory Animals of the National Institutes of Health. The protocol was approved by the University of Wisconsin-Madison Institutional Animal Care and Use Committee (Protocol number: L0037900512). No specific permissions were required for these locations/activities as starlings are not endangered, protected or covered under the Migratory Bird Treaty Act. Twenty male starlings were captured using baited fly in traps on a single farm in Madison, WI (Madison geographic coordinates: latitude, +43°4′12″; longitude, −89°24′0″). After capture, birds were housed indoors in single sex cages (91 cm×47 cm×47 cm), 5 birds to a cage. Food (Purina Mills Start and Grow Sunfresh Recipe, 61S3-IGH-G) and tap water were provided ad libitum. The endocrine state of male starlings is sensitive to photoperiod. Prior to the study, all males were exposed to photoperiods of 18 h light∶6 h dark for at least 8 weeks followed by 8 h light∶16 h dark for 8 weeks. This schedule of long followed by short photoperiods causes starlings to enter a state of photosensitivity that is characteristic of the onset of the fall non-breeding season [43], [44]. In this state, males have regressed gonads, form large flocks, sing high rates of undirected song, and do not display sexual responses or song production in response to females [12]. Males were placed in indoor aviaries (approximately 2 m×2 m×2.5 m) in 5 groups of 4 birds. Each animal was assigned both numbered and colored leg bands for identification. Each aviary was maintained on an 8 h light∶16 h dark photoperiod for the duration of the experiment and contained 4 nest boxes and one multi-perch stand. Food and water were provided ad libitum.

### Conditioned place preference paradigm rationale

To measure song-associated reward, we observed male starlings singing in a home aviary, and then individually transferred each bird to one of the two distinctive compartments of a conditioned place preference (CPP) apparatus (details below), which is referred to as the “song-paired” compartment. The next day, birds were allowed to move freely between the two compartments of the apparatus. If the song-paired compartment was preferred, we considered song-associated CPP to have occurred. The idea is that the neural state triggering or resulting from singing is the unconditioned stimulus. When this neural state is paired with a distinct compartment of a CPP apparatus, this compartment becomes associated with the state associated with the singing behavior. Thus, if song is stimulated or maintained by a pleasurable or rewarding neural state, then males should develop a CPP (method reviewed in [16], [17]). This method is similar to that employed in studies of copulation-induced reward in rats [20], [45] and used here because birds will not sing undirected song when removed from an aviary and placed alone into a CPP apparatus (at least not reliably).

### Song-associated conditioned place preference testing

Ten days after being placed in aviaries, males in each aviary were observed to sing at high rates (as determined based on quantification of song rate for 30 min on 4 days prior to the day of habituation). On day 11, we began the CPP experiment, which includes a habituation, conditioning and preference test. A CPP apparatus consisted of a standard cage (approximately 118 cm×59 cm×59 cm) divided into two distinct compartments by a removable opaque divider. Each side was lined with colored construction paper covering the walls, floor, ceiling and divider side of the cage. One side was decorated with red paper and the other side was decorated with blue. The colors of the left and right sides were counterbalanced across the 4 cages.

Males were first habituated to the apparatus and baseline preferences determined. Specifically, males were placed into a CPP apparatus (with no divider separating the two sides) for 30 min. During this period, the amount of time birds spent on each side was recorded. Males were returned to flocks immediately after the habituation period.

Conditioning was performed the following day. Dividers were inserted to separate the two sides of each CPP apparatus. A single observer positioned behind a one-way glass recorded behavior for each of the 4 males within the home aviary for 30 min. The observer recorded the number of songs produced, the duration of time each male spent singing (secs), as well as bouts of feeding and preening. Separate bouts of behavior were counted when separated by 2 seconds. Immediately following behavioral observations, all 4 birds in a focal aviary were rapidly captured and placed singly into one of the two distinct sides of a separate CPP apparatus (half were confined to the blue side and half were confined to the red side). Birds were confined to the single side for 30 min. No birds were observed to sing during the period of confinement. Following conditioning animals were returned to their home aviaries.

The extent to which birds developed a song-associated preference was tested the following day (testing phase). The divider was removed from each apparatus and each male from an aviary was placed singly into the center of a place preference apparatus. A single observer who was blind to which side the bird had been placed during conditioning measured the time each bird spent on each side of the apparatus for 30 min. Birds did not sing during this period. The final CPP score was calculated as the seconds spent on the previously song-paired side of the CPP apparatus minus the previously measured baseline preference for this side.

To summarize, the birds were allowed to sing in a flock in an aviary, and then afterwards restricted to one of 2 distinctly decorated sides of a conditioned place preference (CPP) apparatus. The next day, during the “testing phase”, birds were allowed to move freely between the two compartments of the apparatus and the amount of time spent on each side was recorded. The idea was that the neural state associated with the preceding singing behavior was the unconditioned stimulus. When this state was paired with one side of the CPP apparatus, if the state was rewarding, then later when given a choice we predicted a bird would display a preference for that side of the apparatus. The birds were never observed singing in the apparatus so it is not possible that they found a place they preferred and then began to sing.

### Tissue collection and processing

**Figure 1:**
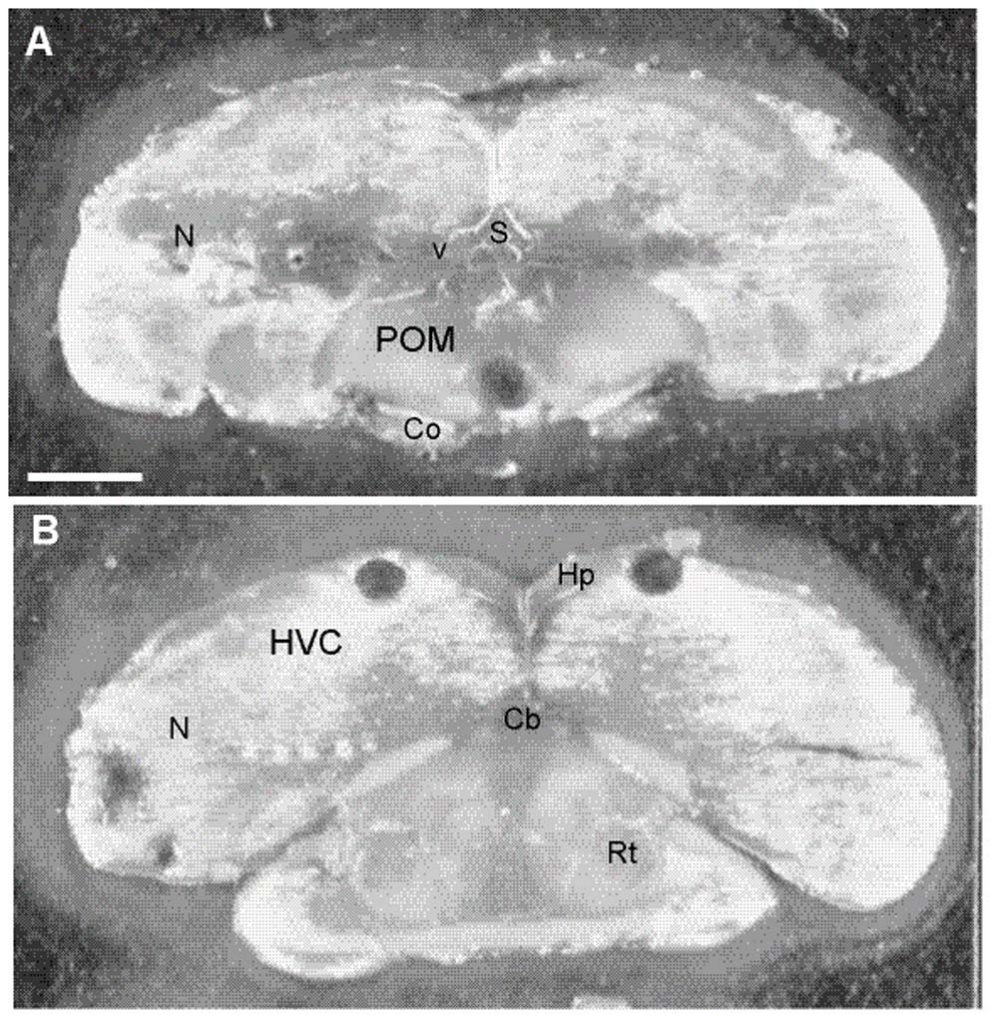
Locations and sizes of tissue samples collected from POM and HVC for qPCR. Photomicrographs of 200 µm thick coronal brain sections from which samples were collected from A) POM and B) HVC. Circular holes are centered within each region of interest and illustrate the location and size of tissue punches. Abbreviations: N =  nidopallium, v =  ventricle, S =  septum, Co =  optic chiasm, Hp =  hippocampus, Cb =  cerebellum, Rt =  nucleus rotundus. See text for additional details.

One day after testing, birds were rapidly decapitated and brains removed and frozen with isopentane on dry ice. We collected neural tissue at this time to gain insight into constitutive mRNA expression in birds associated with their consistent individual tendencies to sing. Individual starlings sang at highly consistent rates across multiple observation days. Song rate measures on each of 5 observation days (i.e., on 4 pre-test days prior to conditioning and on conditioning day) correlated positively with song rates produced on all other days (Pearson r values ranged from 0.70–0.89, p<0.0007 in all cases). This consistency indicates that the patterns of gene expression on the day of tissue collection can be interpreted to reflect the stable tendencies of birds to sing at high or low rates. Brains were wrapped in foil and stored at −80°C. Brains were sectioned at 200 microns and samples of the POM and HVC were collected under a dissecting microscope using a Stoelting brain punch tool (cat. 57401; 1.25 mm punch on two consecutive 200 µm slices, both hemispheres were included in each punch; Fig. 1). Tissue for each brain area for each individual was immediately extruded into separate chilled 1.5 ml microcentrifuge tubes. Slices from which punches were collected were photographed and the locations of brain punches verified. Tissue punches were stored at −80°C.

### RNA extraction and reverse transcription

RNA extraction was completed using the Bio-Rad Aurum Total RNA Fatty and Fibrous Tissue Kit (Catalog No. 732–6830; Bio-Rad, Hercules, CA). Briefly, tissue was homogenized at medium speed using a Dremel tool for 25 seconds. RNA was isolated with PureZOL and treated with DNase according the manufacturer's instructions. RNA was eluted from the columns using 30 µl nuclease free water. RNA concentration was measured with a Nanodrop system (Thermo Scientific, Wilmington, DE).

Conversion to single stranded cDNA was carried out using Invitrogen SuperScript III First-Strand Synthesis System (Catalog No. 18080–051; Life Technologies, Carlsbad, CA) according to manufacturer's instructions with 100 ng starting RNA and oligo(dT) (from kit). Standard cDNA was derived from a pool of all of the samples for the given brain region and was utilized for the genes of interest as well as for the reference genes. Relative gene expression for MOR and PENK in POM and HVC was quantified using qPCR.

### Primer design

NCBI Primer Blast was used to design primers for control reference genes hypoxanthine phosphoribosynltransferase (HPRT) and glyceraldehydes-3-phosphate dehydrogenase (GAPDH) using chicken, *Gallus gallus* sequences (Table 1). The preproenkephalin (PENK) primer was designed using NCBI Primer Blast and the zebra finch genome (Table 1). The mu opioid receptor (MOR) primer was designed based on [46] (Table 1). Netprimer (Premier Biosoft) was used to examine secondary structure of all primers to avoid primer products. Non template controls were run with each primer pair to check for formation of primer-dimers. Primer runs yielded single peak melt curves indicating amplification of single genes. The qPCR reaction product for each gene was sequenced using Sanger sequencing with both forward and reverse primers at the University of Wisconsin Biotechnology Center and sequences are provided in S1 Table. Using NCBI BLAST all sequences match the intended targets.

**Table 1:** Primers used to measure opioid-related mRNA and reference genes.

Gene	Accession #	Direction	Sequence	Ta	Product
Preproenkephalin	NM_001245451.2	Forward	TGTCAGCAAAAGATACGGAGG	58.5°	185 bp
		Reverse	GCAGAATGGAGTCGGAAAG		
Mu Opioid Receptor	XM_002187352	Forward	GCAGATGCCCTTAGCAACAAG	57.0°	165 bp
		Reverse	CACGTAGCGATCCACACTCA		
GAPDH	NM_204305.1	Forward	AGCAATGCTTCCTGCACTAC	57.0°	121 bp
		Reverse	CTGTCTTCTGTGTGGCTGTG		
HPRT	NM_204848.1	Forward	GACCTGGACTTGTTCTGCAT	57.2°	107 bp
		Reverse	ATTTCACGTGCCAGTCTCTC		

### Quantitative real-time PCR (qPCR)

qPCR was performed following the manufacturer's instructions for Sso Fast EvaGreen Supermix (BioRad, 172–5201, Hercules, CA, USA). Both samples and standards were run in triplicate with 20 µl reaction volume. Specificity for each primer pair was confirmed using melt curve analysis. Each plate contained 5 standards at 1∶4 dilution series, beginning at 500 ng/µl and a negative control in which cDNA was replaced by nuclease free water. All runs were performed with an initiation step at 95°C for 30s, followed by 40 cycles including a 5s dissociation phase at 95°C, a 30s annealing phase unless otherwise noted below (annealing temperature (Ta) varied based on primer set), and a 20s elongation phase at 72°C. All plates then went through a melt curve from 60°C to 88°C, 0.5 degrees for each 5s step. Plates were read following each elongation step and each melt curve step. Runs had to achieve efficiency between 90–110% and an R2 of at least 0.990 to be acceptable.

Mean Ct values for each sample (the average cycle number at which each sample triplicate crossed the amplification threshold, which here was set to 200 RFU), were transformed via the Pfaffl Method to yield individual relative expression level values for each gene of interest for each bird [47]. Two reference genes were used for each nucleus.

## Results

Raw data for all of the analyses reported below are available in S2 Table.

Given the prediction that undirected singing behavior and opioid release are tightly coupled (perhaps interacting reciprocally) and related to individual reward state, we predicted that in addition to singing behavior, opioid-related mRNA in the POM would statistically explain CPP. To test this prediction, we ran backward multiple regression analyses with CPP entered as the dependent variable and song rate, a measure of opioid-related mRNA in POM (either PENK or MOR in separate analyses), and non-song measures of eating plus drinking and preening entered as predictor variables. PENK and MOR were examined in separate multiple regression analyses because they were intercorrelated such that inclusion in one model was not appropriate. The same 2 qPCR data points were missing for MOR and PENK measures in POM, reducing the sample size to 18. Two additional qPCR data points were missing for MOR measures in HVC. Thus, including MOR measures in HVC and POM in the same multiple regression analysis would reduce the sample size to 16. Therefore, although for the PENK analysis we included the measure of PENK expression in HVC as another independent variable, we performed a separate analysis to examine MOR in HVC (as described below). Measures of opioid-related mRNA in HVC were included as a control to test our prediction that opioids specifically in POM but not HVC underlie song-associated reward. Finally, the non-song measures were included because we predicted that CPP was specifically induced by song-associated opioid release rather than opioids released by other behaviors such as feeding, drinking or preening. Residual probability plots indicated that data conformed to assumptions of multiple regression analyses.

### Analysis including MOR mRNA

**Figure 2:**
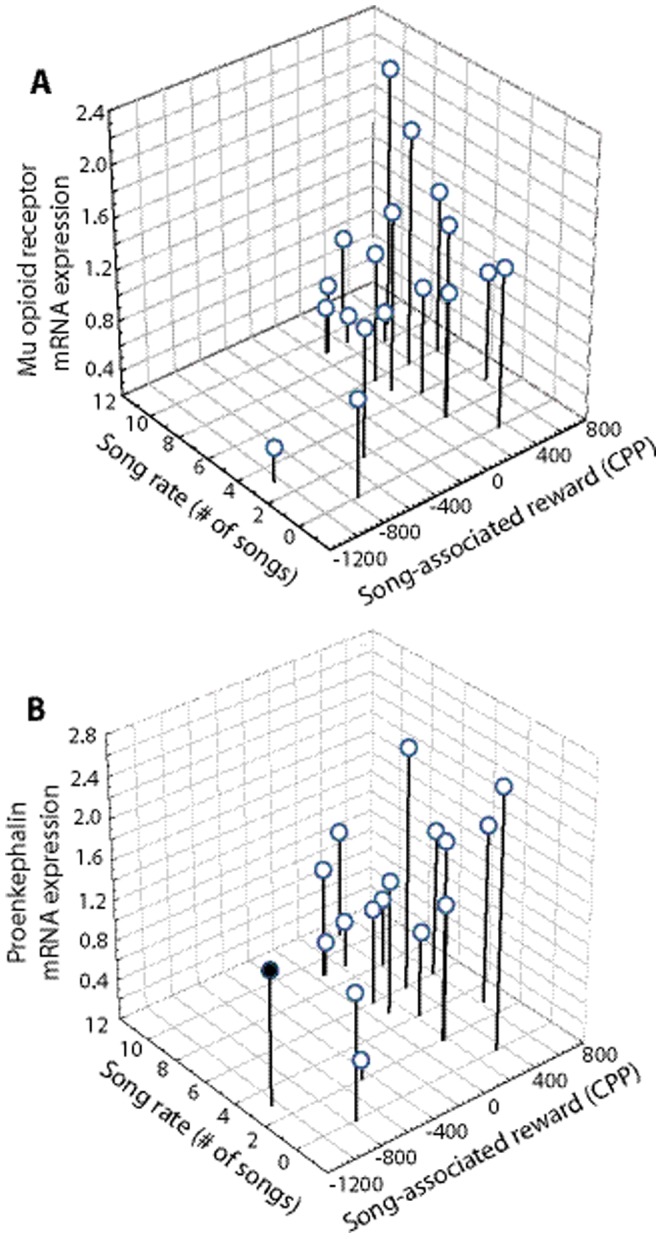
Scatterplots illustrating correlations between undirected singing behavior, opioid-marker mRNA expression in POM, and reward state. For both panels A and B, the Y axis indicates the number of songs produced by male starlings just prior to being placed in one side of a CPP apparatus (the song-paired side). The X axis represents reward associated with singing behavior as reflected in the development of a preference for the previously song-paired side of the apparatus (i.e., secs spent on the previously song-paired side of the apparatus minus secs spent on that side prior to conditioning). The Z axis in panel A represents mu opioid receptor expression. The Z axis in panel B represents preproenkephalin expression. Each point within a panel represents data from a single male. The filled point in panel B was a statistical outlier not included in the analysis. See text for results of multiple regression analyses.

Results of multiple regression analysis with CPP entered as the dependent variable and song rate, MOR in POM, preening, and eating plus drinking entered as predictor variables showed both song rate (Beta  = 0.53, Std. Err. of Beta  = 0.21, t (15)  = 2.56, p = 0.022) and MOR in POM (Beta  = 0.45, Std. Err. of Beta  = 0.21, t (15)  = 2.17, p = 0.047) to statistically explain variance in CPP (n = 18; adj R2 = 0.30, p = 0.027; Fig. 2A). No other variables contributed significantly to the model. The result of the same analysis but with MOR in HVC replacing MOR in POM as an independent variable was not significant (n = 18; adj R2 = 0.13, p = 0.08). For this analysis the only variable close to contributing significantly to variance in CPP was song rate (Beta  = 0.43, Std. Err. of Beta  = 0.23, t (16)  = 1.90, p = 0.08).

### Analysis including PENK mRNA

A multiple regression analysis was run with CPP entered as the dependent variable and song rate, PENK in POM, PENK in HVC, preening, and eating plus drinking entered as predictor variables. One outlier was identified in this analysis based on a standard residual >2* sigma (Fig. 2B). Both song rate and PENK in POM contributed significantly to variance in CPP with or without this outlier. When the outlier was removed preening was additionally found to contribute significantly. We report here results with the outlier removed because the model better fit the data based on the adjusted R2 and standard error. Results indicated that song rate (Beta  = 0.76, Std. Err. of Beta  = 0.18, t (13)  = 4.15, p = 0.001), PENK in POM (Beta  = 0.62, Std. Err. of Beta  = 0.18, t (13)  = 3.38, p = 0.005), and preening (Beta  = 0.39, Std. Err. of Beta  = 0.17, t (13)  = 2.34, p = 0.036) statistically explained variance in CPP (n = 17; adj R2 = 0.57, p = 0.003; Fig. 2B).

## Discussion

Although engaging in behavior with no obvious or immediate function may play important roles in learning and maintaining the behavior, the mechanisms that reinforce or facilitate such behaviors are not clear. Here we examined the hypothesis that male European starlings produce undirected song outside of the breeding season in part because the act of singing in this context is facilitated and/or maintained by opioid-mediated intrinsic reward. Consistent with this hypothesis, we report positive couplings between individual reward state (as reflected in CPP) and undirected singing behavior and both PENK and MOR mRNA expression in the POM.

### Results support a role for opioids in the POM in song-associated reward

Both enkephalin opioids and mu opioid receptor stimulation in the POM induce a positive affective state (as revealed using CPP tests) in rats [37], [38]. The present findings can thus be interpreted to suggest that singing induces opioid receptor and enkephalin expression in the POM and consequent reward. Alternatively the results can be taken to suggest that opioid-mediated reward induces a positive affective state which facilitates singing. We discuss the former possibility first. The idea that vocal production can induce opioid release is supported by studies in ring doves, *Streptopelia risoria*, showing that auditory pathways stimulated by a bird's own vocal behavior are enkephalinergic [48] and studies in humans showing that vocal behaviors (undirected swearing, affiliative social laughter, and vocal repetition or rhythmic respiration [e.g., during meditation]) induce analgesia (an indirect reflection of opioid release [49]–[51]). Similarly, undirected song production in starlings is correlated positively with opioid-mediated analgesia [31]. In addition to promoting opioid release, activation of the vocal-respiratory system is an integral part of techniques (e.g., mantra meditation and laughter) in humans used to promote well-being [52], [53]. It is thus possible that the stimulation of the vocal-respiratory system during undirected song production in starlings facilitates opioid release and positive affect via a well-conserved mechanism. Altogether these studies support the idea that vocal production releases opioids and can promote positive affect. The correlation between opioid-related mRNA in POM and song-associated CPP in the present study further suggests that opioid activity in POM may function to reward singing behavior.

An alternative, and not mutually exclusive, interpretation of the present data is that opioid release in the POM induced by individual or environmental factors (such as the presence of food, safety of a flock or the absence of predators) induces a positive affective state which then facilitates singing behavior. Past studies support the idea that opioid receptor stimulation facilitates undirected vocal behavior. For example, in songbirds the opioid receptor antagonist naloxone blocked undirected singing behavior [30]. In contrast, at least some data suggest that mu opioid receptor agonists facilitate purring behavior and euphoria in cats (e.g., [54]).

We propose that the relationship between undirected singing behavior and an opioid-mediated reward state is likely bidirectional with opioids both initiating and maintaining undirected song production. This idea is supported by studies showing that laughter (in humans and similar vocalizations in rats) as well as other forms of vocal production in humans (e.g., singing behavior) both induce and are facilitated by a positive affective state [55]–[58]. Overall, our data are unique in that they establish a positive linear link between opioid markers in the POM, and reward associated with singing behavior (no matter the direction of the relationship between the reward and song). Our next step in this line of work will be to directly manipulate opioids in the POM to determine causal relationships.

### Relationships are specific to song and the POM

With one exception, measures of non-song behavior (preening and feeding plus drinking) did not significantly contribute to regression models, indicating that variability in CPP was specifically related to song production and not to other behaviors that may also be associated with reward and influence or be influenced by opioid activity [59]–[61]. The exception to this finding was with a multiple regression analysis examining the statistical contributions of feeding plus drinking, preening, and PENK in POM and HVC to variance in CPP. Although preening was not found to contribute significantly to CPP when all data were included in the model, a positive correlation between preening and CPP appeared with removal of a statistical outlier. It is thus possible that preening may release opioids (or be induced by opioids) resulting in a CPP; however, this relationship may also reflect a statistical artifact.

If opioid activity underlies song-associated reward, then we predicted that only brain areas involved in song in which opioids are known to induce reward (such as POM) would relate to CPP. To gain insight into this prediction we included as a negative control in our analyses measures of MOR and PENK mRNA expression in HVC, a song control region that contains enkephalin and mu opioid receptors [46], [62], [63] but plays no known role in reward. Analyses including MOR and PENK mRNA expression in HVC returned no significant models, indicating that relationships between opioids, CPP, and undirected singing behavior are specific to the POM.

### Interpretation of CPP data

The present study represents the third time that a strong, positive, linear correlation has been identified between undirected singing behavior and CPP [14], demonstrating this to be a robust relationship. The CPP methodology used here differs somewhat from CPP tests used in studies of drug or food reward (reviewed [15], [64]) because unlike a food or drug reward we cannot administer the “act of singing” to birds. They either sing or they do not. This means that we cannot pair the act of singing with one side of the apparatus and a lack of singing with the other side of the apparatus an equal numbers of times as is common in studies of food or drug reward. Thus the novelty of the song-paired and unpaired sides of the apparatus cannot be controlled. Although it may be that something about the familiar side of the CPP apparatus is more appealing to birds singing higher rates of undirected song, we do not have a compelling argument in support of this idea. We therefore suggest as in past studies that the most parsimonious interpretation of the results is that undirected singing behavior is tightly coupled to a positive (or a less negative) state. Detailed discussion of this and other interpretational issues can be found in [14].

### What is the function of song-associated reward?

We propose that opioid release in the POM and reward accompanying vocal production function to ensure that important vocal behaviors occur even in the absence of an immediate, obvious behavioral response from an individual recipient. For example, intrinsic reward associated with vocal behavior is likely critical for early vocal learning, when young birds must practice song at high rates in order to develop effective adult song (reviewed in [65], [66]). In adulthood reward associated with vocal production would allow open-ended song learners such as starlings to continue to learn new songs in adulthood, and would allow closed-ended learners such as zebra finches to maintain song structure [66].

We do not suggest that the only function of undirected song is practice or that it is entirely unrelated to the presence of conspecifics. For example, undirected singing behavior may be facilitated by flock membership [67] and serve to maintain flocks [13], which certainly benefits birds by offering protection and opportunities for social learning (e.g., females may gain information used in future mating decisions [68], [69]). There is also evidence that during song learning individuals can incorporate elements into a song repertoire in response to behavioral responses of conspecifics [70]–[72]. We suggest however that during courtship or territory defense the behavioral response of a conspecific to singing behavior (e.g., attracting and copulating with a female) is the primary mechanism that rewards song. In contrast, outside the context of mating or territoriality, we suggest that although the presence of conspecifics may facilitate singing behavior, the act of singing itself may be a primary reinforcer for this type of vocal behavior.

### Conclusions and future directions

The goal of this study was to gain insight into the role of opioids in the POM in song-associated reward. It must be noted however that opioids are not only involved in reward, but also regulate learning and memory, stress, appetite, analgesia, and other physiological processes [73]. It is thus possible that our data reflect a role for opioids in such processes that may then influence relationships between song and CPP. Therefore, a necessary next step in this line of research will be to directly evaluate the role of intra-POM opioid release in song-associated reward by blocking PENK or MOR expression in POM using antisense approaches or by blocking mu opioid receptors in the POM pharmacologically. In addition to the POM, the bed nucleus of the stria terminalis and periaqueductal gray are implicated in undirected singing behavior [40], and along with other areas such as the amygdala, nucleus accumbens, and ventral tegmental area are considered components of an opioid sensitive reinforcement network [74]. Future studies are thus needed to explore a potential role for opioids in these regions in song-associated reward. Finally, the role of other neurochemical systems involved in reward, including neurotensin, glutamate, dopamine, and endocannabinoids as well as other opioids and receptors (e.g., dynorphin, endorphins, kappa, and delta receptors) must be considered in future work.

## Supporting Information

S1 Table **Results of Sanger sequencing of the qPCR reaction product for each gene.**
(XLSX)

S2 Table **Raw data used in reported analyses.**
(XLSX)
